# Increased apoptosis of regulatory T cells in patients with active autoimmune hepatitis

**DOI:** 10.1038/s41419-017-0010-y

**Published:** 2017-12-14

**Authors:** Katharina John, Matthias Hardtke-Wolenski, Elmar Jaeckel, Michael P. Manns, Klaus Schulze-Osthoff, Heike Bantel

**Affiliations:** 1 Department of Gastroenterology, Hepatology and Endocrinology, Hannover Medical School, Hannover, Germany; 2 Department of Molecular Medicine, Interfaculty Institute for Biochemistry, University of Tuebingen, Tuebingen, Germany

Dear Editor,

Autoimmune hepatitis (AIH) is characterized by autoimmune-mediated inflammatory liver injury requiring life-long immunosuppressive therapy. Treatment failure or disease relapse are associated with the risk of disease progression to liver fibrosis and cirrhosis^1^. The pathophysiology of AIH involves the environmental triggering of a liver-specific immune response in genetically susceptible individuals. In addition, an impairment of immunosuppressive regulatory T cells (Tregs) might play a role^2^. Surprisingly, a recent study demonstrated that the frequency of Treg cells in the blood is not reduced in AIH patients compared with healthy subjects^3^. Moreover, patients with active disease revealed even a higher number of Tregs compared with patients in remission^3^. This unexpected observation is in line with another study demonstrating that intrahepatic Tregs are enriched in active AIH^4^. The mechanism underlying the existence of active AIH despite an increased number of functionally intact Tregs remains unclear. Intriguingly, a recent study in mice demonstrated that Tregs are highly sensitive to apoptosis, which was associated with a low expression of the anti-apoptotic molecule c-FLIP and the development of autoimmunity^5^. In the present study, we therefore investigated whether peripheral Tregs from patients with active AIH reveal increased apoptosis and might therefore numerically not be sufficient for disease control despite their increased number.

We first assessed the frequency of Tregs, that is, CD4^+^CD25^high^CD127^low/-^FOXP3^+^ cells, among all CD4^+^ cells (Figs. 1a, d) and found no significant differences between the Treg cell number of healthy controls (7.3 ± 0.7%) and AIH patients with or without biochemical remission (8.6 ± 0.4% and 9.2 ± 0.6%, respectively). We then compared the percentage of Treg cell apoptosis (Figs. 1b, e) and demonstrated that patients with active AIH reveal significantly (*p* < 0.01) more apoptotic Tregs (15.4 ± 1.5%) compared with patients in remission (10.1 ± 1.2%) or healthy controls (6.9 ± 0.9%). No significant difference in Treg cell apoptosis was detected between AIH patients in remission and healthy persons (Fig. 1b). We also analyzed the ratio of apoptosis in Treg and T effector (CD4^+^CD25^low/-^FOXP3^−^) cells (Fig. 1c) and found a significantly (*p* < 0.01) higher rate of Treg versus Teff cell apoptosis in patients with active AIH (15.4 ± 1.5% vs. 12.2 ± 1.6%). These data therefore suggest that increased apoptosis of Tregs might contribute to a disturbed T-cell balance and disease activity in AIH.

**Fig. 1:**
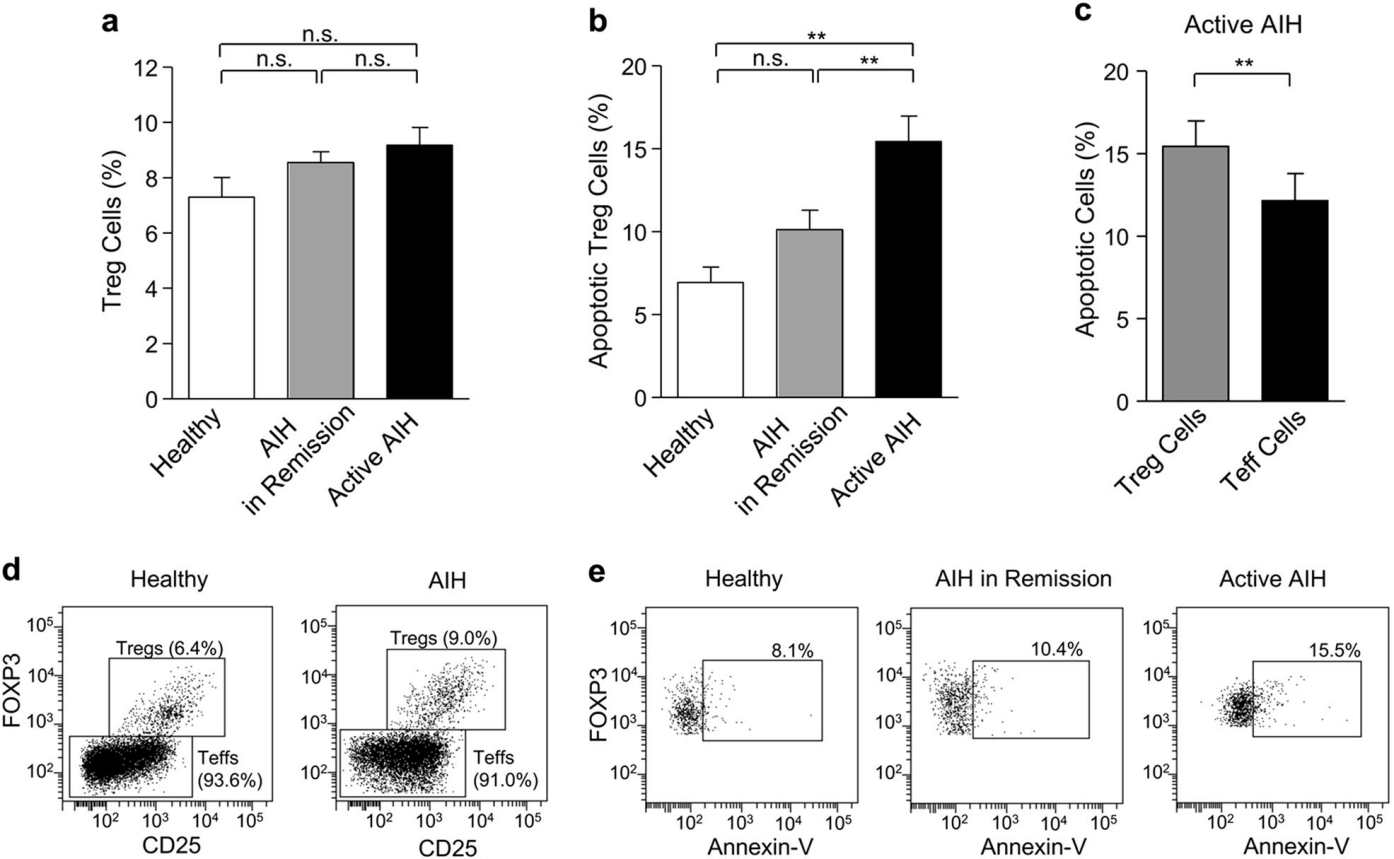
**a** Flow cytometric assessment of the percentage of CD4^+^CD25^high^CD127^low/-^FOXP3^+^ (T regulatory) cells among all CD4^+^ T cells in healthy subjects (*n* = 15) and patients with AIH in remission (*n* = 57) or active AIH (*n* = 42). **b** Analyses of the percentage of apoptotic (annexin-V-positive) Treg cells among the T regulatory cell population in healthy persons (*n* = 15) and AIH patients with (*n* = 57) or without (*n* = 42) biochemical remission. **c** Comparison of the percentage of apoptotic regulatory with apoptotic effector (CD4^+^CD25^low/-^FOXP3^−^) T cells in patients with active AIH (*n* = 42). **d** Representative flow cytometry of a healthy individual (left panel) and a patient with AIH (right panel) indicating the percentage of regulatory and effector T cells among CD4^+^ T cells. **e** Representative flow cytometry of a healthy individual (left panel), a patient with AIH in remission (middle panel) and a patient with active AIH (*right panel*) indicating the percentage of apoptotic Tregs among Treg cell population. Data represent means ± SEM; statistical analyses were performed by Mann–Whitney’s *U*-test (Fig. 1a, b) or Wilcoxon test (Fig. 1c). **p < 0.01; n.s. = not significant. Patient characteristics: AIH patients with biochemical remission had normal aminotransferase levels (AST 27.8 ± 1.0 U/L; ALT 23.9 ± 1.0 U/L), whereas in patients with active AIH the levels were increased (AST 81.0 ± 23.2 U/L; ALT = 92.1 ± 13.0 U/L). Patients with/without biochemical remission were positive for anti-nuclear (ANA), smooth-muscle (SMA), soluble-liver-antigens (SLA) or liver/kidney microsomal type-1 (LKM-1) antibodies in 56.1/66.7%, 33.3/50.0%, 17.5/11.9% and 10.5/9.5% of the cases. Viral hepatitis A-E was excluded. At the time of investigation, 88% (50/57) of the patients with and 95% (40/42) of the patients without remission received standard immunosuppressive therapy. The study was approved by the Ethics Committee of Hannover Medical School.

Tregs account for about 5–10% of CD4^+^ T lymphocytes in healthy individuals and their impairment may cause autoimmune disease^2,6^. However, along with others, we found no reduction or even an increase of Tregs in patients with active AIH compared with healthy subjects^3,4^. Since there is no evidence of impaired Treg cell function in AIH patients^3^, the question remains whether the Treg cell number is insufficient to downregulate AIH activity. In this study, we demonstrated increased apoptosis of Tregs in patients with active AIH compared with those in remission or healthy individuals. We also observed elevated Teff cell apoptosis in active AIH, but to a lower percentage as compared with Treg cell apoptosis. Thus, enhanced Treg cell apoptosis and a resulting insufficient increase of Treg cell number might represent one explanation for the lack of disease control in patients with active AIH. The inflammatory microenvironment could negatively influence the viability of Tregs and might therefore represent a target for therapeutic interventions. In this context, a recent in vitro study showed that interleukin-2 (IL-2) deficiency triggers apoptosis of Tregs from AIH patients^7^. Vice versa, it was demonstrated that IL-2 induces the expression of the anti-apoptotic molecule Mcl-1, thereby contributing to Treg cell survival^8^. Thus, low-dose IL-2 supplementation might represent a novel strategy to protect Tregs from apoptosis in AIH. Further studies are required to identify factors, which contribute to increased Treg cell apoptosis and might represent novel therapeutic targets for AIH.

## References

[1] Czaja AJ, Manns MP (2010). Advances in the diagnosis, pathogenesis, and management of autoimmune hepatitis. Gastroenterology.

[2] Longhi MS (2004). Impairment of CD4+CD25+ regulatory T-cells in autoimmune liver disease. J. Hepatol..

[3] Peiseler M (2012). OXP3+ regulatory T cells in autoimmune hepatitis are fully functional and not reduced in frequency. J. Hepatol..

[4] Taubert R (2014). Intrahepatic regulatory T cells in autoimmune hepatitis are associated with treatment response and depleted with current therapies. J. Hepatol..

[5] Plaza-Sirvent C (2017). c-FLIP expression in Foxp3 expressing cells is essential for survival of regulatory T cells and prevention of autoimmunity. Cell Rep..

[6] Sakaguchi S (2008). Regulatory T cells and immune tolerance. Cell.

[7] Chen YY (2016). Human intrahepatic regulatory T cells are functional, require IL-2 from effector cells for survival, and are susceptible to Fas-ligand-mediated apoptosis. Hepatology.

[8] Pierson W (2013). Antiapoptotic Mcl-1 is critical for the survival and niche-filling capacity of Foxp3^+^ regulatory T cells. Nat. Immunol..

